# Exploring the chemotypic variability of *Silybum marianum* and *Silybum eburneum* by biochemical and genetic characterization

**DOI:** 10.3389/fpls.2025.1584104

**Published:** 2025-06-03

**Authors:** Marianna Pasquariello, Tommaso Martinelli, Roberta Paris, Anna Moschella, Roberto Colombo, Alice Di Bello, Jessica Frigerio, Abdenour Kheloufi, Mohammad Amin Mirzaabolghasemi, Damiano Puglisi, Salvatore Esposito, Stefano Scalercio, Nino Virzì, Pasquale De Vita, Nicola Pecchioni, Laura Bassolino

**Affiliations:** ^1^ Council for Agricultural Research and Economics, Research Centre for Cereal and Industrial Crops (CREA-CI), Bologna, Italy; ^2^ NBFC, National Biodiversity Future Center, Palermo, Italy; ^3^ Council for Agricultural Research and Economics, Research Centre for Plant Protection and Certification (CREA-DC), Firenze, Italy; ^4^ Council for Agricultural Research and Economics, Agriculture and Environment Research Centre (CREA-AA), Bologna, Italy; ^5^ Department of Biotechnology and Biosciences, University of Milano Bicocca, Milan, Italy; ^6^ Department of Ecology and Environment, University of Batna 2, Batna, Algeria; ^7^ Section of Plant Breeding and Biotechnology, Department of Horticulture, Faculty of Agriculture, Vali-e-Asr University of Rafsanjan, Rafsanjan, Iran; ^8^ Council for Agricultural Research and Economics, Research Centre for Cereal and Industrial Crops (CREA-CI), Foggia, Italy; ^9^ CNR-IBBR, National Research Council of Italy, Institute of Biosciences and Bioresources, Research Division, Portici, Italy; ^10^ Council for Agricultural Research and Economics, Research Centre for Forestry and Wood (CREA-FL), Rende, Italy; ^11^ Council for Agricultural Research and Economics, Research Centre for Cereal and Industrial Crops (CREA-CI), Acireale, CT, Italy

**Keywords:** flavonolignan, chemotype, milk thistle, isosilychristin, germplasm (genetic) resources

## Abstract

The *Silybum* genus belonging to the Asteraceae family, is composed of two species, *marianum* and *eburneum*, although, in the past, their classification was not always appropriate. While *Silybum marianum* is very well known since ancient times for the medicinal properties of a blend of different flavonolignans contained in the achenes and named silymarin, very little information is available about *Silybum eburneum* chemodiversity. Here, we describe the biochemical characterization of a wide *ex situ* germplasm collection including 83 wild *Silybum* accessions collected during *ad hoc* sampling campaigns in Italy, Spain, Iran and Algeria as well as accessions acquired by seed GenBanks and studied at both population and single plant level. Interestingly, our results confirm the presence of only three chemotypes in *S. marianum*, namely A, B and C. Conversely, *S. eburneum* accessions, exhibit a distinct and stable chemotype (D) where isosilychristin is the predominant silymarin component. Additionally, DNA barcoding based on the ribosomal DNA region *ITS2* combined with morphological phenotyping and chemotyping, successfully resolves frequently found mistakes in the identification of the two species. These findings significantly expand our knowledge of the global biodiversity of the *Silybum* genus and provide valuable insights for future breeding programs and potential applications in nutrition and human health sciences.

## 1 Introduction

The *Silybum* genus is grouped within the tribe Asterales of the Asteraceae family and according to Tutin et al. (1976) comprises only two species: *Silybum marianum* (L.) Gaerth, commonly known as milk thistle, and *Silybum eburneum* Coss. and Durieu, commonly named elephant thistle. The geographic origin of this genus remains unknown due to a lack of information on its speciation (Goeden, 1976). However, most reports list *S. eburneum* as mainly located in the North and East Africa area (Morocco, Algeria, Tunisia and Kenya) (Willkomm, 1870) and in Spain (Polunin and Smythies, 1973), while *S. marianum* is widely distributed across North Africa, Europe and Central Asia. Subsequently, *S. marianum* has been naturalized and also cultivated as a crop in many countries including Japan, sub-Saharan Africa, North and South America, Australia and New Zealand (POWO Plants of the World Online Facilitated by the Royal Botanic Gardens, Kew, 2023). Both *S. marianum* and *S. eburneum* are wild spinescent herbaceous plants, annual or biennial, and they are considered synanthropic species, meaning they are often linked to human activities (Tutin et al., 1976). These two species are closely related, but possess at least two distinct traits for clear identification: (i) cauline leaf spines length (up to 8 mm in *S. marianum*; 7 to 15 mm in *S. eburneum*) and (ii) outer involucral bracts phenotype (with appendage tapering into a recurved spine in *S. marianum*; without spine in *S. eburneum*) (Tutin et al., 1976). Contrary to what previously stated by Hetz et al. (1995), the absence of leaf variegation is not a peculiar feature of *S. eburneum*. Also, Puglisi et al. (2024) recently conducted a DArT analysis, showing that an accession previously misclassified as *S. eburneum* based solely on the absence of leaf variegation was actually identical to *S. marianum* accessions. Regarding the utilization of these two species, *S. marianum* has been used for medicinal purposes for over 2000 years, mainly to treat liver diseases, as well as for liver protection from toxic substances (Morazzoni and Bombardelli, 1995; Gillessen and Schmidt, 2020). The therapeutic effects of *S. marianum* are closely linked to the accumulation of an isomeric mixture of bioactive flavonolignans known as silymarin, which is found in the pericarp and fruit coat (Cappelletti et al., 1984). Silymarin content usually ranges from 1 to 4.3% of the achene weight (Martin et al., 2006; Andrzejewska and Sadowska, 2008) and its main constituents are silybin (diastereoisomers A and B; SILA, SILB) (*syn.* silibinin, silybinin), isosilybin (diastereoisomers A and B; ISOA, ISOB), silychristin (SILYC) and silydianin (SILYD) (Lee et al., 2007), along with the minor constituents such as silychristin B and isosilychristin (ISOSILYC) (Kaloga, 1981; Smith et al., 2005). Silybin is often the most abundant flavonolignan in medicinal preparations and frequently considered the most bioactive ingredient of silymarin (Bijak, 2017) although research has demonstrated that each flavonolignan possesses specific biological activity (Polyak et al., 2010; Biedermann et al., 2016). The pharmacological effects of silymarin include anti-inflammatory, antioxidant, anti-cancer, hypoglycemic, neuroprotective, and immunomodulatory properties (Zhang et al., 2024).

Silymarin profiles in *S. marianum*, assessed at single plant level by Martinelli et al., 2017, have been classified into two stable chemotypes, A and B, based on the relative concentration of flavonolignans. In particular, chemotype A is characterized by high levels of both silychristin and silybin, whereas chemotype B mainly contains silydianin (Martinelli et al., 2017). A third chemotype, namely chemotype C, shows an intermediate SILYD content and is the result of hybridization between chemotypes A and B (Martinelli et al., 2021). Conversely, in wild populations, which frequently comprise plants with different chemotypes, variable relative flavonolignans content is often observed (Martinelli et al., 2021). While total silymarin content is significantly influenced by various physiological and environmental factors (Martin et al., 2006; Afshar et al., 2015), chemotypes, defined as the relative proportion of flavonolignans, show an extremely high phenotypic stability (Martinelli et al., 2021, 2017). The biosynthetic process leading to chemotype differentiation is genetically regulated and is strictly controlled by a monogenic heritable factor (Martinelli et al., 2023). Consequently, it is fundamental to characterize *S. marianum* chemotypes at single plant level and, subsequently, assess chemotypes frequency at population level.

Very few studies are available on *S. eburneum*. Recently, the mineral, organic acid, free sugar, protein, and phytochemical contents, as well as the antioxidant potentials of various plant organs of both *S. marianum* and *S. eburneum* have been investigated (Maaloul et al., 2024b), highlighting some differences between the two species. Indeed, seed oils of both species were found to be rich in unsaturated fatty acids, phenolic compounds, and tocopherols, which are all associated with high antioxidant activities, suggesting the important potential of *Silybum* organs as sources of nutrients for producing functional foods (Maaloul et al., 2024a). However, *S. eburneum* seed oil demonstrated the greatest antioxidant activity. Regarding flavonolignans composition, *S. eburneum* silymarin was initially described as mainly composed of silybin and silydianin (Halbach and Winkler, 1971; Carreras, 1975; Szilagyi and Tetenyi, 1978). However, more recently (Adzet et al., 1993), reported a high isosilychristin content in seeds of a single *S. eburneum* accession derived from Spain.

The present work aims to characterize the silymarin composition and chemotype identification of *Silybum* genus accessions at both single plant and population level, collected from a wide range of locations within the species’ native geographical cradle. Additionally, we describe for the first time the silymarin composition of *S. eburneum* fruits from different origins aiming for a detailed chemodiversity assessment. Moreover, we employed DNA barcoding to resolve the ongoing debate regarding the *S. marianum*/*S. eburneum* differentiation which is crucial for species identification and expanding nutritional and health related applications.

## 2 Materials and methods

### 2.1 Plant material and open field cultivation

The *ex situ* collection used in the present work consists of 83 accessions, 9 of which belong to *S. eburneum* and 74 to *S. marianum* species (**Supplementary Table S1**). The number of *S. eburneum* accessions is lower in comparison to *S. marianum* because the geographic area where this species is native to is smaller and the disparity of sample numerosity between the two species is representative of this difference. Italian, Algerian, and Iranian accessions were collected in wild areas during *ad hoc* sampling campaigns during the spring - summer seasons of 2023 and 2024, according to the ESCONET protocol (2009). For these materials, fruits were harvested from wild populations, with accession representing an independent population. Only accessions separated by at least 5 Km from other individuals or isolated by geographical barriers (e.g. mountains, sea, rivers) were collected. Sampling was performed when fruits were fully developed, and plants were at phenological stage 86 and 89 according to the BBCH scale (Biologische Bundesantalt, Bundessortenamt, and Chemische Industrie) for *S. marianum* (Martinelli et al., 2015). The remaining accessions were retrieved from various GenBank germplasm collections as reported in **Supplementary Table S1**, where origin, collection site and collection date are reported (if available). Accessions were named in an arbitrary code according to the country, the region or the Genebank FAO code from which seeds were retrieved. Two accessions of *S. marianum* chosen for the contrasting chemotype, namely G23 for chem A and G20 for chem B, and two accessions of *S. eburneum* selected because of the different geographic origin (Polit1 form Spain and ALG3 from Algeria) were sown in Bologna (Italy) (44°31’56.2”N 11°21’05.8”E) in October 2023 and cultivated in open field conditions as described by (Martinelli et al., 2023) for phenotypic evaluation. Three plants for accession were planted and a completely randomized block design was used for the field experimental layout. Differences between the species were assessed according to (Tutin et al., 1976) and further used for the identification of wild *S. marianum* and *S. eburneum* accessions.

Additionally, five plants of both five *S. marianum* and five *S. eburneum* were cultivated in a Controlled Environment Room (CER) with long photoperiod (18h/6h light/dark), and leaf samples were collected at two-leaf stage (BBCH stage 12) for molecular barcoding analysis. *S.marianum* accessions were chosen, as chemotype representatives, among the Italian collection due to higher seed availability, whereas *S. eburneum* accessions were selected as representatives of all the geographic origins in the collection.

### 2.2 Silymarin extraction

Silymarin extraction protocol was adapted from Martinelli et al. (2016). Fruits of *S. marianum* and *S. eburneum* were ground with a coffee grinder (Moulinex MC300132) for 1 min. Both fruit pool (FP) and single fruit (SF) extractions were conducted. Fifteen fruits were pooled in FP extraction and 50–55 mg of fruit flour was then taken to the next step. Fruit flour was then added to 1.5 ml of hexane (≥99%) and extracted under constant agitation for 30 min at room temperature. After 30 min centrifugation (14000 rpm), supernatant was removed and, to ensure complete defatting, pellet was extracted a second time overnight with 1.5 ml hexane. After 30 min centrifugation (14000 rpm), the supernatant was discarded and the pellet allowed to dry in a fume hood. Silymarin was finally extracted from the pellet adding 1.5 ml 75% methanol and incubating overnight under constant agitation at room temperature. Silymarin extracts were then stored at −20°C before HPLC analysis.

### 2.3 Silymarin HPLC analysis

High Performance Liquid Chromatography (HPLC) analysis of silymarin was performed on a Dionex Ultimate 3000 system (Thermo Scientific) equipped with a C18 column (Kinetex 2.6 µm, 100 A, 100 by 3 mm; Phenomenex). The HPLC settings were as follows: flow of 0.340 ml min^−1^; column temperature at 23°C; and ultraviolet–visible (UV-Vis) detector at 288 nm. The mobile phase was composed of methanol (Phase A) and 0.1% formic acid (Phase B) using 33% phase A isocratic from 0 to 3 min; 33 to 47% phase A gradient from 3 to 17 min; then 47% phase A isocratic from 17 to 32 min. Flavonolignans and taxifolin identification and quantification were obtained using purified standards (Sigma-Aldrich). For quantification of taxifolin (TAX; typical retention time (RT)= 6.2 min), silychristin (SILYC; RT=14.5 min), silydianin (SILYD; RT= 15.2 min), silybin A (SILA; RT= 22.9 min), silybin B (SILB; RT= 24.0 min), isosilybin A (ISOA; RT= 27.5 min, and isosilybin (ISOB; RT= 28.5 min), a *4 points* calibration curve was created using the following dilutions: 50, 25, 6.25 and 3.125 ug/ml. Isosilychristin (ISOSILYC) concentrations were, instead, determined by peak area relative to the silychristin calibration curve. Using the HPLC protocol described here, SILYC, SILYD, SILA, SILB, ISOA, and ISOB peaks could be separated at the baseline level. In samples where a high SILYD to SILYC ratio was identified, the baseline separation was not possible for these two molecules, and the SILYC peak was integrated using the skim method, as SILYD peak was tailed in standard preparations.

Relative flavonolignans composition of *Silybum* accessions was calculated as the mean of each flavonolignan content in all the accessions with the same chemotype and expressed as percentage of the total silymarin content. Total silymarin content (SILTOT) was calculated as the sum of each flavonolignan, excluding the silymarin precursor taxifolin.

### 2.4 Liquid chromatography-mass spectrometry

Liquid chromatography (LC) has been performed on a LC Vanquish system (Thermo Scientific) using a biphenyl column (Kinetex 1.7 µm 100 x 2.1 mm, Phenomenex). The mobile phase comprised ammonium formate 4 mM in water (Phase A) and ammonium formate 4 mM in methanol (Phase B), both solutions containing 0.1% formic acid. The LC settings were as follows: from 0 to 17 min 30% of Phase B, from 17 min to 32 min 50% of Phase B, and then from 32 min to 36min 30% of Phase B. A constant flow of 0.300 mL/min was applied.

Mass spectrometry analysis of *S. eburneum* silymarin samples was performed using a high-resolution mass spectrometer Orbitrap Exploris 120 (Thermo Fisher).

The ionization source was operated in both positive and negative ionization modes (ESI+; ESI–). Data were acquired using full MS followed by DDA MS2 at resolution 60.000 (full MS) and 15,000 (MS2), respectively. The HCD (Higher Energy Collision Dissociation) method was performed using 10, 20, and 30% normalized collision energies to obtain the M-H and M+H spectra of C_25_H_22_O_10_.

### 2.5 DNA barcoding analysis

Five samples of *S. eburneum* and five of *S. marianum* were retrieved from the *ex situ Silybum* germplasm collection of the CREA-CI (Bologna) and are marked in red in **Supplementary Table S1**. DNA was extracted from young leaves (BBCH stage 12) using the DNeasy Plant Kit (QIAGEN, Milan, Italy), following the manufacturer’s protocol. The purified gDNA was then quantified and assessed for purity using a Qubit 2 Fluorometer and the Qubit dsDNA HS Assay Kit (Invitrogen, Carlsbad, CA, USA). For species identification, the standard markers of DNA barcoding were selected (*rbcL*, *matK*, *psbA-trnH*) together with the Internal Transcribed Spacer (*ITS2*) region, following the protocol described by Cheng et al. (2016). PCR amplification was carried out using Wonder Taq Polymerase (EuroClone S.p.A., Milan, Italy) in a 25 μl reaction volume, according to manufacturer’s instructions. Each reaction contained 1 μl of 10 mM primers, 3 μl of DNA template (25 ng/μl), and the primers described by Gorini et al. (2023). The PCR cycling conditions consisted of an initial denaturation at 95°C for 5 min, followed by 35 cycles of denaturation (45 sec at 95°C), annealing (45 sec at 55°C), extension (1 min at 72°C), and a final extension at 72°C for 7 min. The amplified products were sequenced at Macrogen Europe (Milan, Italy). The sequences were manually edited, primers were removed, and using AliView software 1.28 (Larsson, 2014). The sequences of *S. eburneum* and *S. marianum* were compared to assess genetic variability between the two species. Finally, species identification was conducted through a standard BLASTn comparison against the GenBank database (https://blast.ncbi.nlm.nih.gov/Blast.cgi, accessed on 31 Jan 2025). Taxonomic assignment of each barcode sequence followed the method outlined by Frigerio et al. (2021). Finally, all sequences were submitted to the BOLD System (https://www.boldsystems.org/). NGPhylogeny.fr (accessed on 31 Jan 2025) was used for the Neighbor-Joining tree creation (Lemoine et al., 2019). The Neighbor-Joining tree was created using *ITS2* marker with default parameters, due to its high variability (Cheng et al., 2016).

### 2.6 Statistical analysis

Statistically significant differences between flavonolignans relative content and total silymarin content of the different chemotypes were assessed using Spss software (v 17.0) using one way ANOVA and Tukey HSD for *post hoc* test. Before analysis percentage data were transformed to arcsine square root.

## 3 Results and discussion

### 3.1 Establishment of the *ex situ Silybum* collection

**Figure 1:**
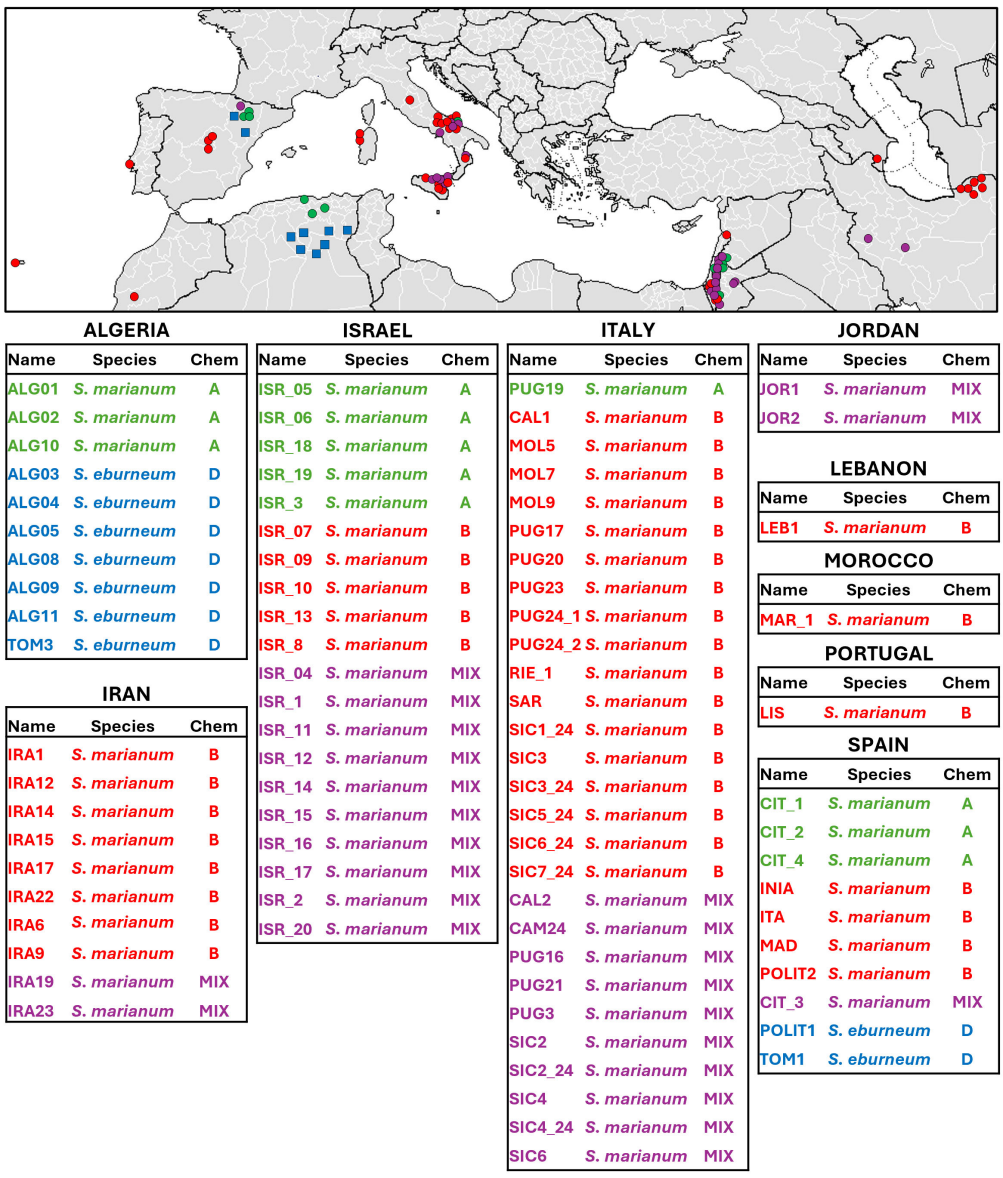
Geographical distribution of the *Silybum* collection. Each dot represents an accession, with colors indicating chemotype classification: green for Chemotype A, red for Chemotype B, blue for Chemotype D, and purple for mixed chemotypes and shape indicating the species: Circle for *S. marianum* and square for *S. eburneum*. Detailed accession localization can be viewed at the following link: https://www.google.com/maps/d/edit?mid=1OnLjRiLyl7NXbvb912lHGnDzwkSot90&usp=sharing. When precise localization was unknown, the accession was localized in the capital city of the Country.

Silymarin composition is a distinctive phytochemical trait of the *Silybum* genus. Chemotypes classification is commonly based on the relative proportion of different flavonolignans with silydianin biosynthesis proposed as the limiting component to define the three diverse chemotypes described to date (Martinelli et al., 2017). To the best of our knowledge, chemodiversity characterization within the *Silybum* genus has so far been limited to *S. marianum* species and previous works were focused on accessions derived from narrow geographical areas (AbouZid et al., 2016; Arampatzis et al., 2019; Martinelli et al., 2021) or from regions where *Silybum* is not native to (Martin et al., 2006; Martinelli et al., 2016; Shim et al., 2024). In this work, aiming to represent the widest biodiversity of the *Silybum* genus, we include accessions from both *S. marianum* and *S. eburneum* in the native geographical cradle. A total of 83 accessions were analyzed, including 28 from Italy, 11 from Algeria and 10 from Iran. These accessions were collected from the wild during *ad hoc* collection sampling campaigns (Material and Methods 2.1). In addition, an *ex situ* germplasm collection was established from various GenBanks, including 34 other accessions not previously studied, to determine the chemical composition of fruits derived extracts and chemotypes distribution across the collection. Each accession is representative of an independent *Silybum* spp. wild population. The complete list of accessions, along with detailed description of the sampling sites and geographical origin, is presented in **Supplementary Table S1** and **Figure 1**. A more detailed map of collection site is also available at the following link: https://www.google.com/maps/d/edit?mid=1OnLjRiLyl7NXbvb912lHGnDzwkSot90&ll=41.44884759871708%2C11.42578403829971&z=6.

### 3.2 Chemodiversity of *Silybum marianum* accessions

Flavonolignan composition of silymarin was analyzed on a total of 292 samples (227 SF and 65 FP) corresponding to 74 *S. marianum* accessions. 61 accessions were first analyzed as FP, each constituted by 15 seeds, and then, depending on seed availability, a variable number of samples were also analyzed as SF, to confirm chemotype identification. For the remaining 13 accessions, seed quantity was a limiting factor and silymarin analysis was performed directly on SF, using as many as possible (See **Supplementary Table S1** for seed quantity used).

**Figure 2:**
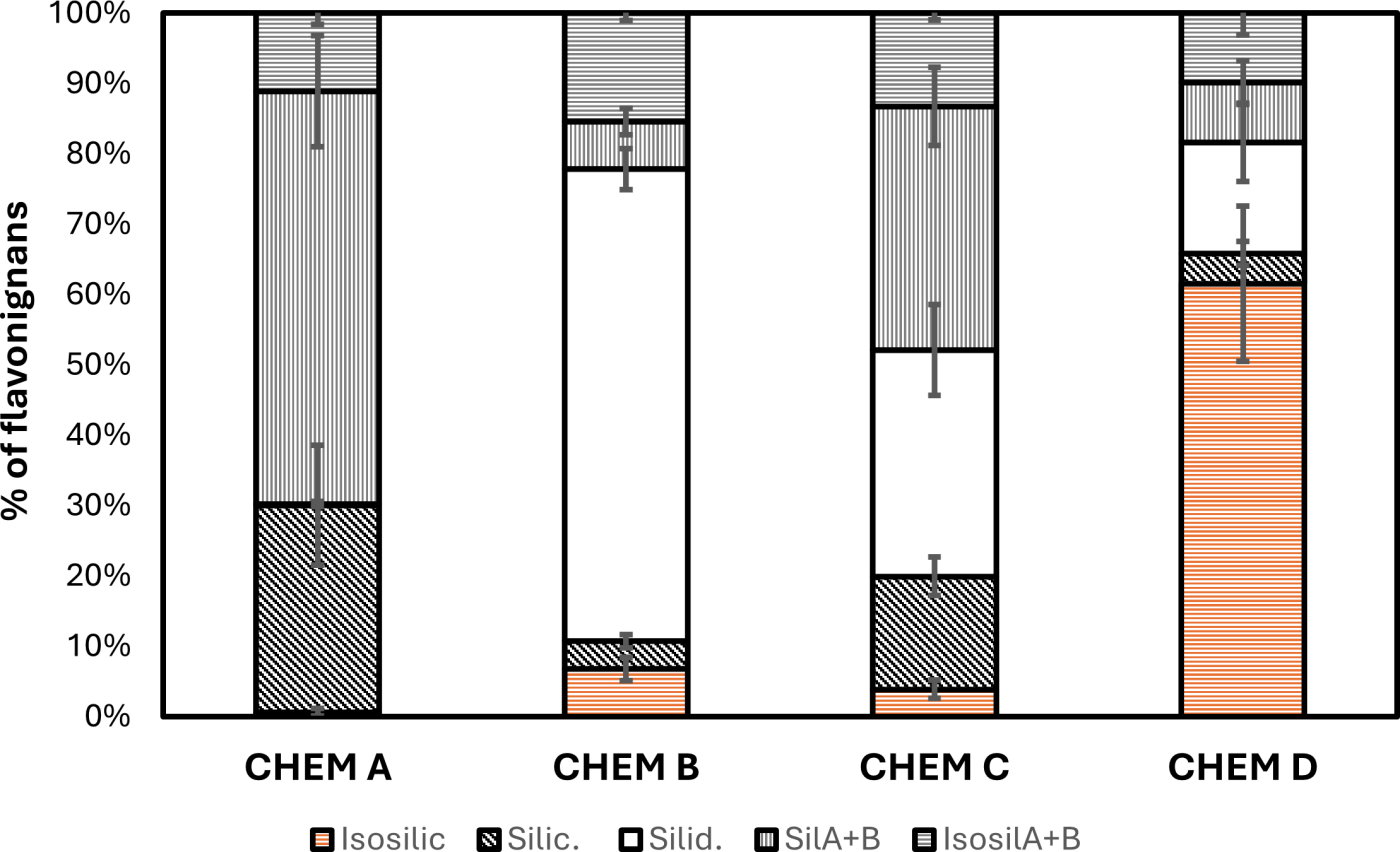
Relative flavonolignans composition of *Silybum* accessions. Data are calculated as the mean of each flavonolignan content in all the accessions with the same chemotype and are expressed as percentage of the total silymarin content. Error bars represent standard deviation of each flavonolignan content.

At population level, 12 FP showed a stable silymarin profile, with an average composition of 29.46% SILYC and 58.67% total silybin (SILA + SILB) characteristic of chemotype A, as described by Martinelli et al. (2017). In contrast, 36 accessions were characterized by a high SILYD relative content (67%), typical of chemotype B (**Table 1**, **Figure 2**). The remaining 13 FP pools had an intermediate silymarin profile.

**Table 1:** Total silymarin and flavonolignan composition of the four chemotypes expressed as a percentage of total silymarin content.

Chemotype	Species	n	Total silymarin	ISOSILYC	SILYC	SILYD	SILA	SILB	ISOA	ISOB
			(mg g fruit DW^-1^)			(% of total silymarin)				
**CHEM A**	*S. marianum*	**87**	**32.79**	**0.59 d**	**29.46 a**	**0.16 d**	**24.06 a**	**34.62 a**	**8.36 abc**	**2.76 d**
SD			13.27	0.53	8.51	0.34	4.70	6.43	1.29	0.57
**CHEM B**	*S. marianum*	**156**	**32.85**	**6.77 b**	**3.96 cd**	**67.09 a**	**2.42 cd**	**4.33 c**	**8.57 abc**	**6.86 a**
SD			10.79	1.65	0.93	2.93	0.85	1.17	0.62	0.60
**CHEM C**	*S. marianum*	**34**	**38.28**	**3.88 c**	**16.03 b**	**32.19 b**	**13.88 b**	**20.75 b**	**8.30 abc**	**4.88 bc**
SD			14.70	1.32	2.73	6.47	1.92	4.01	0.64	1.12
**CHEM D**	*S. eburneum*	**37**	**31.99**	**61.53 a**	**4.34 cd**	**15.77 c**	**2.62 cd**	**2.62 d**	**5.08 d**	**4.79 bc**
SD			18.14	11.02	1.72	5.56	1.04	2.30	1.85	1.57
	*p value*		*ns*	***	***	***	***	***	***	***

Chemotypes are indicated in bold. SD, Standard Deviation; n, number of samples analyzed; ns, not statistically significant; ***p < 0;001; ISOSILYC, Isosilychristin; SILYC, Silychristin; SILYD, Silydianin; SILA, Silybin A; SILB, Silybin B; ISOA, Isosilybin A; ISOB, Isosilybin B; Letters indicate statistically significant differences within each column according to Tukey HSD *post hoc* test.

SF analysis was then performed on the latter 13 accessions with an intermediate silymarin profile and on the 13 accessions with limited number of seed available. Importantly, SF can be always grouped into three chemotypes (A, B, and C), based on the relative proportions of silymarin isomers However, only 34 SF, were classified as chemotype C, showing an average composition of 16.03% SILYC, 32.19% SILYD and 34.63% for total silybin (SILA + SILB), characteristic of chemotype C (**Table 1**). As previously observed the relative content of ISOA is the same between chemotype A, B and C suggesting a common silymarin biosynthetic pathway in these three chemotypes (Martinelli et al., 2017). Interestingly a statistically significant and lower ISOA content was measured in D chemotype suggesting major differences of the biosynthetic mechanism leading to the different flavonolignans in this chemotype. Moreover, when analyzed at the SF level, populations with intermediate silymarin profiles were composed of plants exhibiting chemotype A, B or C, whereas chemotype C plants were never found within populations containing only A or B chemotypes (**Supplementary Table S1**). This result confirms previous findings, which were restricted to Italian populations, that chemotype C is a hybrid between chemotypes A and B (Martinelli et al., 2021). Further, we observed that the number of chemotype C single plants was rather variable among accessions. While the overall percentage of chemotype C single plants is around 13%, when investigated at single population level, values vary from 20% to 60% (data not shown). It is known that chemotype C is the result of hybridization between A and B chemotypes and that chemotype differentiation is a genetically regulated process strictly controlled by a monogenic heritable factor (Martinelli et al., 2023). So, when chemotype C is observed in a SF sample, this implies that the original plant must have been heterozygous for the gene/genes regulating chemotype differentiation. Although the number of individuals analyzed per population in our data is not statistically meaningful, we can assume that the reported 2% outcrossing rate, due to the autogamous nature of *S. marianum* (Hetz et al., 1995), is underestimated or significantly affected by environmental conditions.

Regarding the total silymarin content, the average quantity was 32.7 ± 10.8 mg g DW^-1^, with some variability, as indicated by the standard deviation values. This variation is likely due to the fact that most of the accessions studied here were collected from the wild and grown in different environmental conditions. Indeed, like other secondary metabolites, silymarin accumulation is strongly influenced by the environmental factors (Martin et al., 2006; Afshar et al., 2014, 2015; Shim et al., 2024).

**Figure 3:**
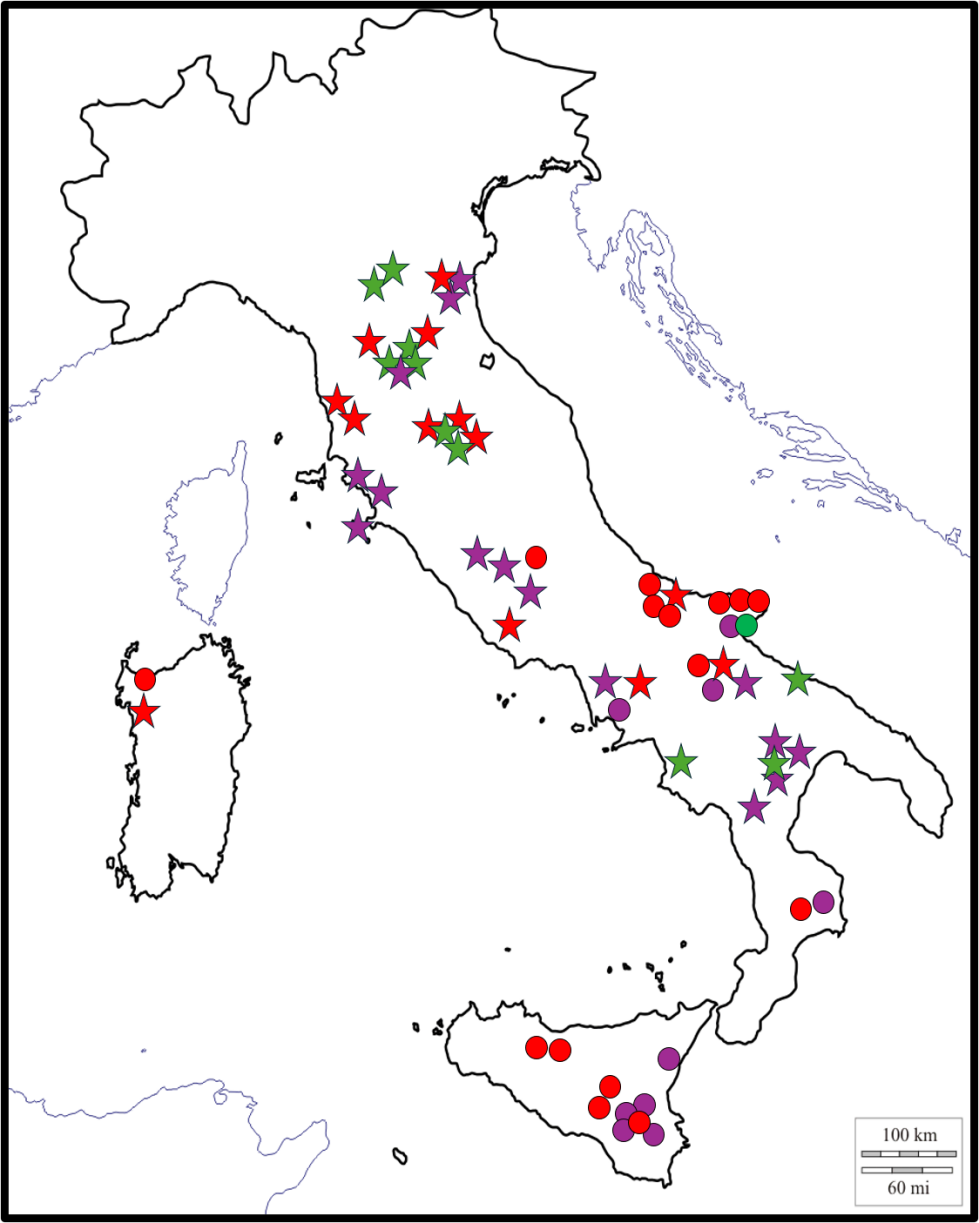
Geographical distribution of Italian accessions. Chemotypes distribution along the peninsula doesn’t show clear geographical clustering. Stars represent accessions collected by (Martinelli et al., 2021); while dots are accessions collected in the present study. Colors represent chemotype classification: green is chemotype A, red is chemotype B and purple is chemotype MIX. Figure modified from (Martinelli et al., 2021).

To further characterize the Italian Mediterranean biodiversity of *S. marianum*, Italian accessions were sampled from southern regions that had not been previously represented in the collection described by Martinelli et al. (2021). Considering the data in **Table 1** alongside with previously reported findings (Martinelli et al., 2021), no clear geographical clustering of chemotypes along the Italian peninsula was observed, confirming the high biodiversity in regions where the species is native (**Figure 3**). The concomitant presence of different chemotypes is also confirmed in the countries where more observations were available (Iran, Israel, and Spain). Overall, the percentage of populations composed of plants with A, B or mixed chemotypes was 16.4, 49.3 and 34.3%, respectively. If both populations with A and B profiles derive from geographic isolation of individuals derived from mixed populations a roughly similar percentage of A and B populations would be expected. The fact that B chemotype is more frequent (almost 3-fold) might indicate a better adaptability of this chemotype to the different environmental conditions. Nevertheless, better adaptability/productivity of plants with B chemotype was not observed under field conditions (personal observation). Alternatively, if B biosynthetic pathway is the complete pathway and A chemotype derives form a mutation of B biosynthetic pathway as hypothesized in Martinelli et al., 2017, it is possible that mixed populations are B populations where the mutation occurred. In this instance populations with only A chemotypes could derive from single plants derived from mixed populations. In this hypothesis the observed frequency of A, B and mixed populations would be meaningful but an extremely frequent mutation rate of the factor causing A chemotype should be hypothesized because A chemotype is present worldwide mostly and in association with B chemotype. Nevertheless, this hypothesis could explain the observed geographic distribution of A, B and mixed populations which is not characteristic of any specific area.

### 3.3 Silymarin characterization of *Silybum eburneum* accessions

**Figure 4:**
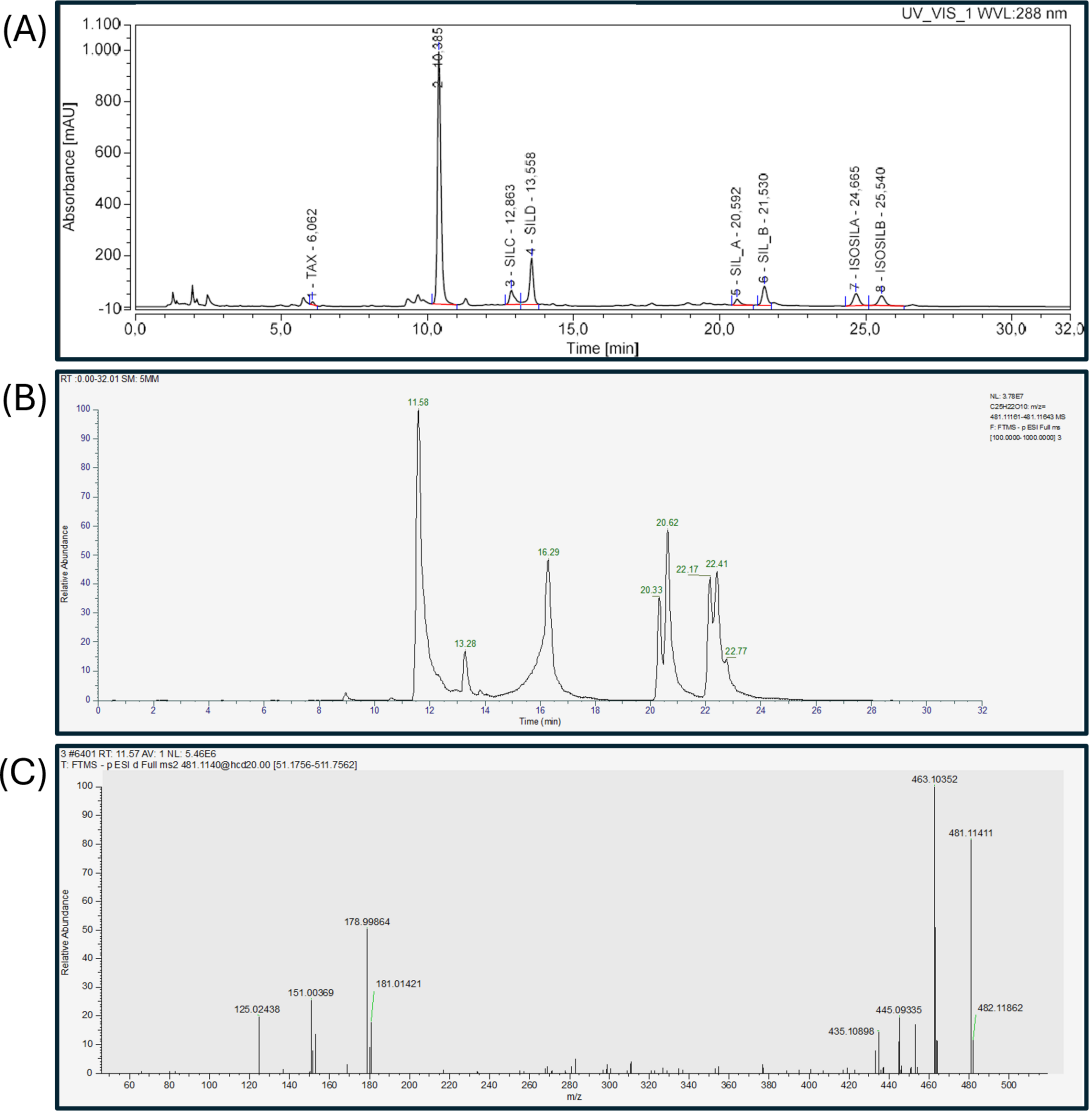
Identification process of the unknown flavonolignan present in all *S. eburneum* accessions. **(A)** UHPLC silymarin separation of a *S. eburneum* accession. The unidentified flavonolignan peaks at 10.422 min. **(B, C)** LC-MS separation of the same *S. eburneum* silymarin extract. **(B)** Chromatographic separation identified all flavonolignans seen by UHPLC, including the C_25_H_22_O_10_ compound peaking at 11.60 in negative polarity, respectively. **(C)** Mass spectrometry of the unknown peak shows main fragments at m/z 125, 151, 153, 178, 181, 435, 445, 453, 463 and 481.

The *ex situ Silybum* collection described here comprises 9 accessions of *S. eburneum* collected in different regions of Algeria and Spain. The silymarin composition of these accessions was analyzed using UHPLC at both FP and SS levels, for a total of 43 samples. Our results showed that, regardless of geographical origin and/or environmental conditions, the HPLC chromatographic profile of all *S. eburneum* accessions is characterized by high levels of a flavonolignan compound, which account for 61,53% of total silymarin. This compound does not correspond to any of our standards and has an elution time of 10.4 min (**Table 1** and **Figure 4A**). All the other flavonolignans were present in lower amounts, with silydianin being the most abundant (15,77%) (**Table 1**; **Figure 4A**). Henceforward, this flavonolignan profile will be referred as chemotype D.

A flavonolignans combination similar to chemotype D was previously reported by Adzet et al. (1993) where the unknown flavonolignan was identified as isosilychristin. Since, to the best of our knowledge, the previous finding has never been further investigated, we performed an LC-MS analysis on silymarin extracted from four *S. eburneum* accessions to confirm the identity of the unknown compound. The LC-MS data confirmed the presence of the same compounds detected by UV-Vis detector. In particular, the peak of interest had a retention time of 11.60 min in both positive and negative polarity, respectively (**Figure 4B**; **Supplementary Figure S1**). When MS mass spectrometry analysis was performed in negative polarity, the unknown compound fragmented into the key ions: 125, 151, 153, 178, 181, 435, 445, 453, 463 and 481m/z (**Figure 4C**). This fragmentation pattern matches isosylchristin which as previously described by Kuki et al. (2012).

Isosylichristin is a structural isomer of silychristin, the second most abundant flavonolignan of silymarin in chemotype A. Although a few reports have explored its biological role, some reports suggest potential pharmacological applications. For example, Biedermann et al. (2016) demonstrated that, isosilychristin, along with other tested stereoisomers, has a stronger antioxidant activity than silybin while remaining not cytotoxic. Moreover, Viktorová et al. (2019) found that isosilychristin can modulate multidrug resistance by downregulating the expression of the transmembrane efflux pump P-glycoprotein (P-gp). Our data indicate that *S. eburneum* achenes contain up to ca 19.5 mg/g DW^-1^ of isosilychristin (expressed as SILYC equivalents; data not shown), corresponding to 61.53% of total silymarin. Considering that different flavonolignans may provide different hepatoprotection (Polyak et al., 2010) and that, flavonolignans isomers, may be competitive for specific cellular targets, the high isosilychristin content in *S. eburneum* silymarin makes it a valuable source for isosilychristin extraction. Further studies are needed to evaluate how its biological activity varies depending on the assay conditions. According to Polyak et al. (2010), silybin A and silybin B are considered the most bioactive isomers of silymarin, therefore chemotype A is the most commercially exploited. However, other flavonolignans like isosilychristin may have unexplored biologically active properties and the availability of new genetic resources of other underutilized chemotypes (B, C and D) is of particular interest for the development of new drugs and pharmacologically active preparations.

Taking as a whole, our results shed light on the chemical composition of silymarin in *S. eburneum* revealing isosylichristin as the primary flavonolignan of chemotype D consistently present across all the studied *S. eburneum* accessions with different geographic origins.

### 3.4 Phenotypic characterization of *Silybum* species and DNA barcoding analysis

To finally clarify the previous identification mistakes regarding *S. marianum* and *S. eburneum*, a phenotypic and molecular characterization of the two species was performed. Hetz et al. (1995), despite quoting the accepted botanical description (Tutin et al., 1976), stated that the difference between the two species is determined by leaf variegation, generating errors in further identifications.

**Figure 5:**
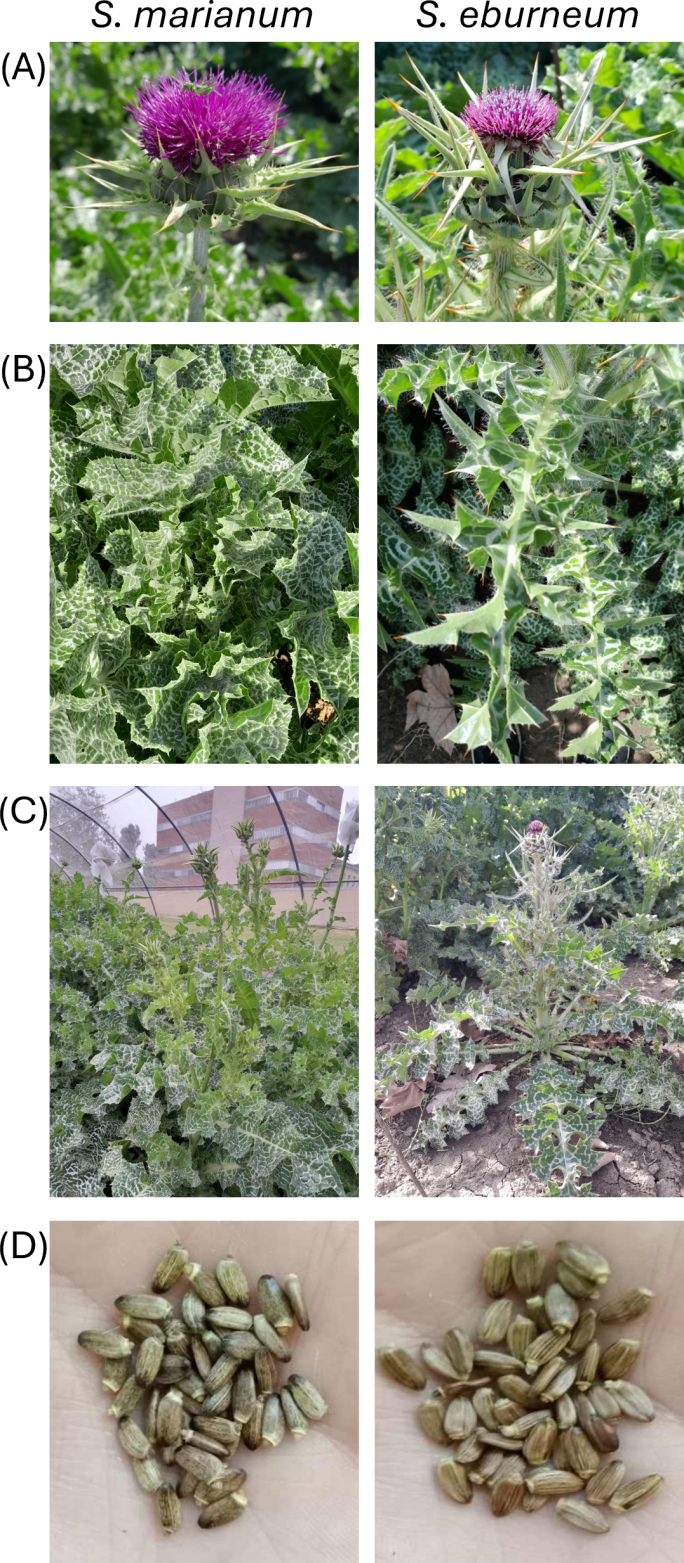
*S. marianum* and *S. eburneum* plants. **(A)** Flowers; **(B)** Leaves; **(C)** whole adult plant; **(D)** achenes.

In order to better characterize the two species, four accessions of both species were grown in an open field experiment and morphological observation of plant phenotype was carried out. **Figure 5** provides a comparative analysis of flowers, leaves, whole plant and achenes. Consistent with the botanical description of Tutin et al. (1976), *S. eburneum* flowers possess longer spines whereas their outer involucral bracts are spineless. In contrast, *S. marianum* flower heads feature an appendage tapering into a recurved spine (**Figure 5A**). Both species basal leaves are pinnatifid but *S. eburneum* leaves are smaller, more hispid and more deeply divided with longer yellowish-brown spines (**Figure 5B**). More importantly the studied *S. eburneum* accessions exhibited variegated leaves, contradicting previous claims by Hetz et al. (1995) and confirming that leaf variegation is not a distinctive trait. Under Northern Italy growing conditions, *S. eburneum* plants were considerably smaller (less than 1 m in height) compared to *S. marianum*, which grew much taller (**Figure 5C**). No differences were spotted in the achenes color and shape between the two species (**Figure 5D**).

Aiming to undoubtedly identify the *S. marianum* and *S. eburneum* accessions used for the phenotyping and more importantly to define a reliable molecular method for the two species identification, a DNA barcoding experiment was performed, targeting all the standard DNA barcoding regions for plants (*rbcL, matK*, *psbA-trnH* and ITS2). A high DNA extraction yield was obtained from the samples ranging from 20–40 ng/µL. Each barcode sequence was taxonomically assigned by using BLASTn analysis (Camacho et al., 2009), matching plant taxa with the nearest identities (maximum identity >99% and query coverage of 100%), confirming specie-level identification. Results, summarized in **Table 2**, show that DNA barcode markers *rbcL, psbA-trnH* and *ITS2* successfully distinguished the *S. marianum* and *S. eburneum*, providing a reliable molecular approach for the accurate identification of these two species. Furthermore, the sequences of *S. eburneum* for the matK and psbA-trnH markers have been deposited in the BOLD System database (https://www.boldsystems.org/) for the first time and ID numbers are reported in **Table 2**.

**Table 2:** DNA barcoding results.

Sample ID	Declared species	Resulted species	BOLD process ID (rbcL, matK, psbA-trnH, ITS2)
SIC1	*S. marianum*	*S. marianum*	CARDO001-25
MOL5	*S. marianum*	*S. marianum*	CARDO002-25
PU19	*S. marianum*	*S. marianum*	CARDO003-25
CAM24	*S. marianum*	*S. marianum*	CARDO004-25
CAL1	*S. marianum*	*S. marianum*	CARDO005-25
POLIT1	*S. eburneum*	*S. eburneum*	CARDO006-25
ALG3	*S. eburneum*	*S. eburneum*	CARDO007-25
ALG4	*S. eburneum*	*S. eburneum*	CARDO008-25
ALG7	*S. eburneum*	*S. eburneum*	CARDO009-25
ALG11	*S. eburneum*	*S. eburneum*	CARDO010-25

In the table are shown sample ID, declared species, resulted species and process ID in BOLD system for the barcode marker *rbcL, matK, psbA-trnH* and *ITS2*.

**Figure 6:**
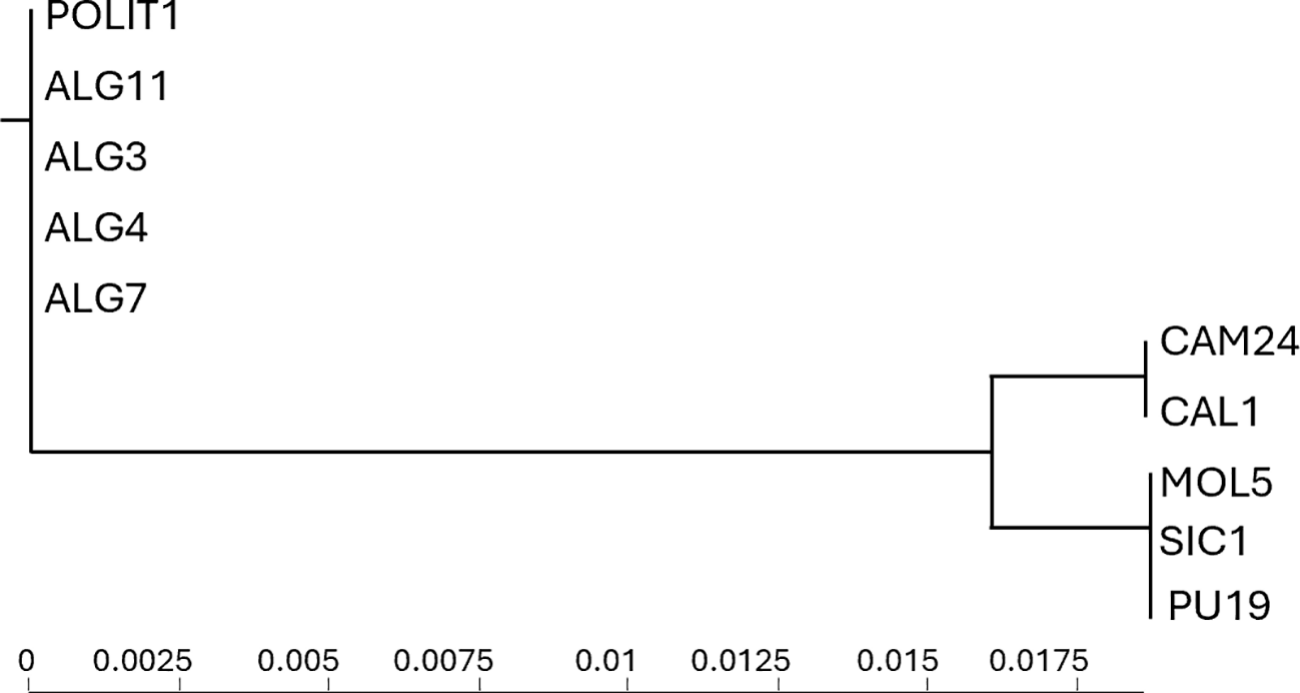
Neighbor-Joining tree depicting the evolutionary relationships among *S. eburneum* and *S. marianum* populations based on genetic divergence.

The Neighbor-Joining tree presented in **Figure 6** illustrates the evolutionary relationships between *S. eburneum* and *S. marianum*. The tree is structured based on genetic divergence, with branch lengths representing evolutionary distances. *S. eburneum* samples originate from Spain and Algeria, whereas *S. marianum* is represented by populations from various Italian regions, including Campania, Calabria, Molise, Sicily, and Puglia. The clear separation of *S. eburneum* and *S. marianum* into distinct clades supports their genetic differentiation. This distinction reinforces their classification as separate evolutionary units, confirming the phylogenetic validity of their taxonomic separation.

## 4 Conclusions

In this study, we present the chemical characterization of a large *ex situ* germplasm collection of the *Silybum* genus. Our data confirm that, despite covering a wide geographical range, representing most of the genus native cradle, only three chemotypes (namely A, B and C) were identified in *S. marianum* accessions. Additionally, in all *S. eburneum* accessions analyzed to date, only one silymarin chemotype D was identified, with isosilychristin as the predominant flavonolignan. These findings expand current knowledge of the global biodiversity of the *Silybum* genus and provide valuable insights for future breeding programs.

To resolve the classifications mistakes between the two species frequently found in literature, we performed a phenotypic characterization of representative accessions of both S. *eburneum* and *S. marianum*. Our analysis identified at least two distinctive morphological traits for clear species identification: (i) flower head and (ii) leaf phenotype. Additionally, considering that all *S. eburneum* accessions studied so far shared the same chemotype (Chemotype D), silymarin chemical composition can be used as a further distinctive trait to distinguish *S. eburneum* from *S. marianum* accessions. Finally, we demonstrated that DNA barcoding based on the *ITS2* gene is a useful, reliable and *cost-effective* methodology to distinguish *S. marianum* from *S. eburneum* accessions. This is a valuable result not only for confirming past and future species identification but also for unlocking new opportunities in the utilization of *Silybum* species.

Overall, these results open new perspectives for the exploitation of *Silybum* derived bioactive compounds with potential applications in the nutritional and health related industries.

## Data Availability

The datasets presented in this study can be found in online repositories. The names of the repository/repositories and accession number(s) can be found in the article/**Supplementary Material**.
