# Supplementary figures and images for: Exploring the chemotypic variability of Silybum marianum and Silybum eburneum by biochemical and genetic characterization
Source: Front Plant Sci. 2025 Jun 3;16:1584104. doi: 10.3389/fpls.2025.1584104 (PMC12188448; doi:10.3389/fpls.2025.1584104)

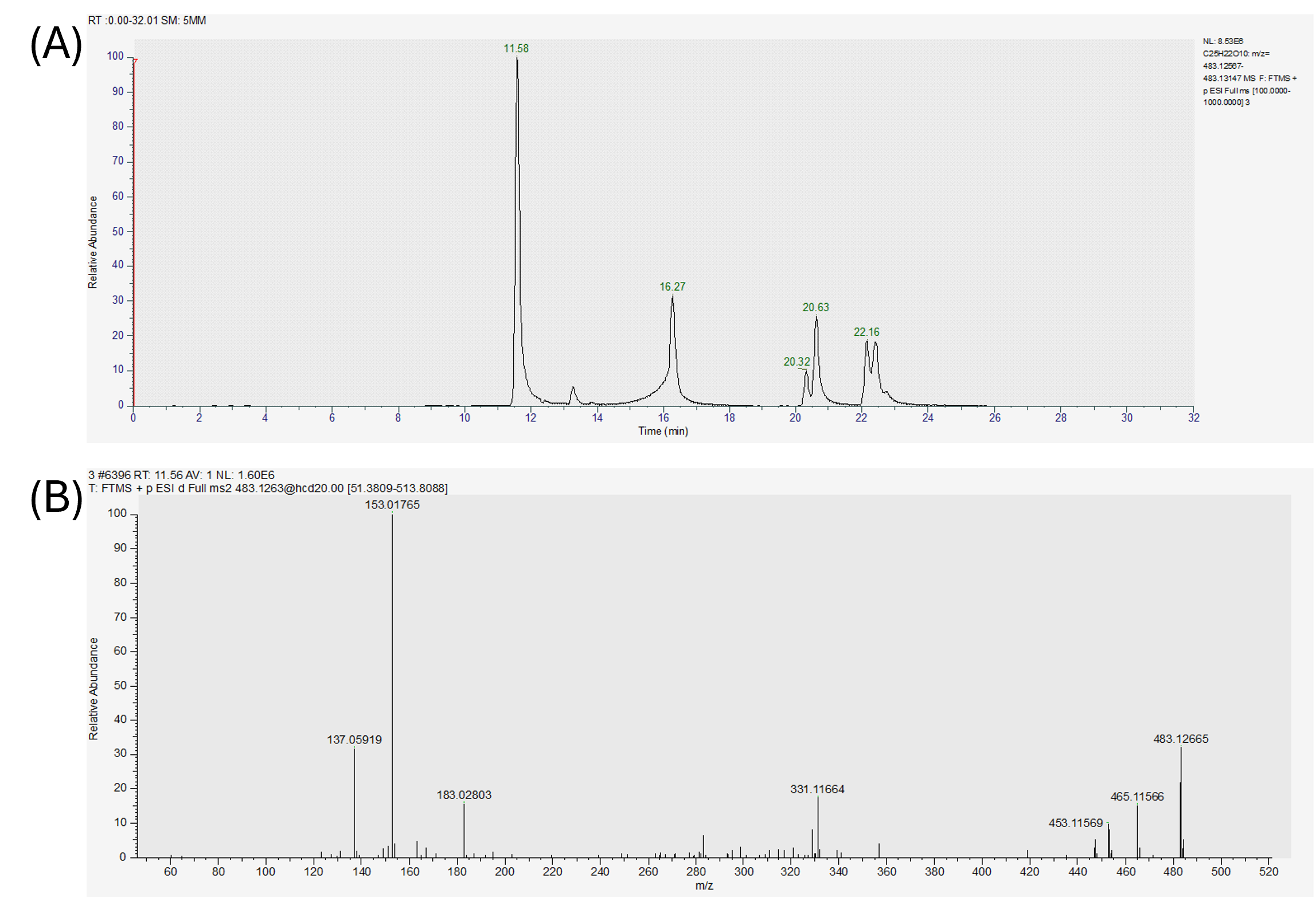

Supplement: Supplementary file 1 [file Image1.tif]
